# Emergency Medicine Student End-of-Rotation Examinations: Where Are We Now?

**DOI:** 10.5811/westjem.2017.10.35144

**Published:** 2017-12-05

**Authors:** Emily S. Miller, Corey Heitz, Linette Ross, Michael S. Beeson

**Affiliations:** * Harvard Medical School, Department of Emergency Medicine, Boston, Massachusetts; † Virginia Tech Carilion School of Medicine, Department of Emergency Medicine, Roanoke, Virginia; ‡ National Board of Medical Examiners, Psychometrics and Data Analysis, Philadelphia, Pennsylvania; § Cleveland Clinic Akron General, Department of Emergency Medicine, Akron, Ohio

## BACKGROUND

Assessment of medical students following the completion of clerkships often involves administration of an examination.^1^,^2^ Before 2011 there was no nationally available, standardized examination for students completing emergency medicine (EM) rotations, and EM clerkship directors (CDs) likely used examinations developed within their own institutions. Significant progress has been made and there are currently several options available to CDs for assessment of students completing EM rotations, including the National EM M4 examinations, Version 1 (V1) and Version 2 (V2), and the National Board of Medical Examiners (NBME^®^) EM Advanced Clinical Examination (ACE).

## OBJECTIVES

This review is a descriptive summary of the development of these examinations and their relevant usage and performance data. In particular, we describe how examination content was edited to affect desired changes in examination performance data and offer a model for educators seeking to develop their own examinations.

## CURRICULAR DESIGN

In 2011 the Clerkship Directors in Emergency Medicine (CDEM) developed the first nationally available, standardized examination to assess fourth-year medical students (M4) completing an EM rotation.^3^ This examination, the National EM M4 examination, consists of 50 multiple-choice questions written according to the NBME^®^ item-writing guidelines^4^ and assesses topics in a published EM M4 curriculum.^5^,^6^ A second comparable version of this examination was released in 2012.^7^ Both versions were expanded to 55 questions in 2015 and are updated annually by CDEM.

National EM M4 examination performance is reviewed annually including student scores, item difficulty (p-value), the percent of students answering a question correctly, and item discrimination. Examination developers aimed for a broad range of difficulty of questions, reflected by a broad range in scores, with a target mean examination score of 80% correct. The mean score of V1 of the National EM M4 examination has ranged from 76.5–81.9 with standard deviation (SD) 3.6–4.6, from its implementation in 2011 through 2017. The fluctuation in examination means is attributed in large part to annual edits to the examination. For example, in 2015 six questions with p-values > 0.95 were revised to generate more difficult questions, and the mean score dropped appropriately from 81.5 (SD 3.7) to 78.2 (SD 4.2).^8^ The mean score of V2 was 72.1 (SD 4.0) in 2012, the first year it was available. Four of the 50 questions had p-values < 0.3 and were revised.^9^ Subsequently, from 2013–2017 the mean examination score ranged from 77.3–82.1 (SD 3.8–4.9).

The point biserial correlation (r_pb_) is a measure of item discrimination and reflects how well a question distinguishes a student who performed well on the examination from those who did not. The r_pb_ ranges from −1 to 1 with positive values indicating a positive correlation and values > 0.2 considered reasonably good.^10^,^11^ The average r_pb_ for V1 questions ranged from 0.201–0.217 from 2011–2017. The average r_pb_ for V2 questions was 0.196 in 2012, the first year it was available, and improved to 0.234 the following year after nine questions with r_pb_ < 0.2 were revised to improve performance.^9^ The range of r_pb_ for V2 questions since revisions in 2013 until 2017 has been 0.234–0.258.

These examples of examination modification demonstrate the ability to edit underperforming items to more closely align with desired examination performance metrics. Educators seeking to develop examinations could employ similar techniques.

## IMPACT

The table shows the number of examination administrations and the number of clerkships using the examinations. In 2016–2017, 72 clerkships from 69 U.S. medical schools used V1, and 48 clerkships from 43 U.S. medical schools used V2.

The NBME® provides examinations to assess students completing clerkships in many disciplines. In 2013, NBME® released its first examination for assessment of EM students. The NBME^®^ EM ACE was developed by a taskforce of CDEM members and NBME^®^ staff to assess content in the same published curriculum assessed by the National EM M4 examinations. The EM ACE consists of 100 multiple-choice items. Scores are equated across forms and are scaled to have a mean score of 70 and SD of 8. Of the 145 U.S. medical schools accredited by the Liaison Committee on Medical Education (LCME), 56 (39%) used the EM ACE in 2016–2017.

In 2015, the NBME^®^ conducted a webcast, standard-setting study to develop grading guidelines for the EM ACE. The recommended range for minimum passing score was 53–62 and for honors score was 74–91.^12^ Of note, the NBME^®^ charges a per-examination fee for use of the EM ACE, whereas the National EM M4 examinations are freely available to CDs from LCME-accredited medical schools.

In summary, in the past six years, several end-of-rotation examination options for EM M4s have become available and are being widely used. The National EM M3 examination, also developed by CDEM, was scheduled for release July 1, 2017 (M. Tews, personal communication). These examinations help fill a void in assessment of EM students.

## Figures and Tables

**Table:** Usage of the National EM M4 examinations (Version 1 and Version 2) and the NBME® EM ACE since implementation.

	Number of examination administrations			Number of clerkships administering examination		
		
Academic year	V1	V2	ACE	V1	V2	ACE
2011–12	1,828	n/a^	n/a	20	n/a	n/a
2012–13	3,229	576	n/a	48	48	n/a
2013–14	2,718	534	3,844	46	42	50
2014–15	2,216	606	4,721	47	52	45
2015–16	2,745	955	5,260	66	48	57
2016–17	2,847	1,128	5,231	72	48	60
Total	15,583	3,799	19,056			

*ACE*, Advanced Clinical Examination.

* Indicates the academic year from July 1 through the following June 30 for all years except 2016–17, which is through June 1.

^ “n/a” indicates the examination was not available for those dates.
